# Genomic Analysis of a Highly Virulent NDM-1-Producing *Escherichia coli* ST162 Infecting a Pygmy Sperm Whale (*Kogia breviceps*) in South America

**DOI:** 10.3389/fmicb.2022.915375

**Published:** 2022-06-10

**Authors:** Fábio P. Sellera, Brenda Cardoso, Danny Fuentes-Castillo, Fernanda Esposito, Elder Sano, Herrison Fontana, Bruna Fuga, Daphne W. Goldberg, Lourdes A. V. Seabra, Marzia Antonelli, Sandro Sandri, Cristiane K. M. Kolesnikovas, Nilton Lincopan

**Affiliations:** ^1^Department of Internal Medicine, School of Veterinary Medicine and Animal Science, University of São Paulo, São Paulo, Brazil; ^2^One Health Brazilian Resistance Project (OneBR), São Paulo, Brazil; ^3^School of Veterinary Medicine, Metropolitan University of Santos, Santos, Brazil; ^4^Department of Microbiology, Instituto de Ciências Biomédicas, Universidade de São Paulo, São Paulo, Brazil; ^5^Departamento de Patología y Medicina Preventiva, Facultad de Ciencias Veterinarias, Universidad de Concepción, Chillán, Chile; ^6^Department of Clinical Analysis, School of Pharmacy, University of São Paulo, São Paulo, Brazil; ^7^R3 Animal, Florianópolis, Brazil

**Keywords:** carbapenems, NDM carbapenemases, nosocomial bacteria, one health, wildlife, aquatic environment

## Abstract

Carbapenemase-producing Enterobacterales are rapidly spreading and adapting to different environments beyond hospital settings. During COVID-19 lockdown, a carbapenem-resistant NDM-1-positive *Escherichia coli* isolate (BA01 strain) was recovered from a pygmy sperm whale (*Kogia breviceps*), which was found stranded on the southern coast of Brazil. BA01 strain belonged to the global sequence type (ST) 162 and carried the *bla*_NDM–1_, besides other medically important antimicrobial resistance genes. Additionally, genes associated with resistance to heavy metals, biocides, and glyphosate were also detected. Halophilic behavior (tolerance to > 10% NaCl) of BA01 strain was confirmed by tolerance tests of NaCl minimal inhibitory concentration, whereas halotolerance associated genes *katE* and *nhaA*, which encodes for catalase and Na^+^/H^+^ antiporter cytoplasmic membrane, respectively, were *in silico* confirmed. Phylogenomics clustered BA01 with poultry- and human-associated ST162 lineages circulating in European and Asian countries. Important virulence genes, including the *astA* (a gene encoding an enterotoxin associated with human and animal infections) were detected, whereas *in vivo* experiments using the *Galleria mellonella* infection model confirmed the virulent behavior of the BA01 strain. WHO critical priority carbapenemase-producing pathogens in coastal water are an emerging threat that deserves the urgent need to assess the role of the aquatic environment in its global epidemiology.

## Introduction

The rapid and global spread of carbapenemase-producing Enterobacterales has triggered an unprecedented public health crisis due to the lack of novel clinically effective antibiotics (Queenan and Bush, 2007; Nordmann et al., 2011a; Lee et al., 2022). Carbapenems are broad-spectrum β-lactam antibiotics that have been administered as a last-line resort, being generally reserved to treat life-threatening infections caused by multidrug-resistant (MDR) Gram-negative bacterial infections (Papp-Wallace et al., 2011; Lee et al., 2022). The production of carbapenemases by certain Enterobacterales and non-fermentative bacteria, can threaten the efficacy of these antimicrobials making them useless (Queenan and Bush, 2007; Nordmann et al., 2011a; Lee et al., 2022).

Due to their clinical impacts in human medicine, carbapenemase-producing bacteria were recent classified as critical priority pathogens by the World Health Organization (WHO) (Tacconelli et al., 2018). Particularly, the emergence of metallo-β-lactamase NDM-1-producing bacteria has been a phenomenon of global interest (Nordmann et al., 2011a,b; Dortet et al., 2014). Of epidemiological concern, although the successful spread of plasmids encoding *bla*_NDM_-type genes has been primarily related to nosocomial settings (Wu et al., 2019), there is growing evidence of their occurrence beyond the human medicine context (Mills and Lee, 2019; Ranjan and Thatikonda, 2021). Indeed, NDM-1-producing bacteria have been increasingly reported in environmental samples (mostly in anthropogenic-impacted aquatic environments) (Ranjan and Thatikonda, 2021). More critically, NDM-1-positive bacteria have begun to be documented in wild animals (Fischer et al., 2013; Liao et al., 2019; Mairi et al., 2020), which could indicate, in part, that these critical-priority bacteria can spill over into natural ecosystems, and then further spread in wildlife (Dolejska and Literak, 2019; Mills and Lee, 2019; Cohen et al., 2020; Mairi et al., 2020).

In this study, we report the identification of an NDM-1-positive *Escherichia coli* strain belonging to the international clone sequence type (ST) 162 in a pygmy sperm whale (*Kogia breviceps*), highlighting negative clinical and ecological implications related to the dissemination of WHO critical priority pathogens in the marine environment.

## Materials and Methods

### Bacterial Isolation, Identification, and Antimicrobial Susceptibility Testing

During a surveillance study conducted to investigate the occurrence and genomic features of critical priority Gram-negative pathogens circulating at the marine ecosystems in Brazil, part of the Grand Challenges Explorations—New Approaches to Characterize the Global Burden of Antimicrobial Resistance program, we characterized a multidrug-resistant *E. coli* recovered from a pygmy sperm whale (*Kogia breviceps*), during the COVID-19 lockdown.

The animal (Supplementary Figure 1) was received at CEPRAM/R3 Animal (Florianópolis, Santa Catarina state, Southern Brazil), as part of the Santos Basin Beach Monitoring Project, licensed by the Brazilian Institute of the Environment and Renewable Natural Resources (IBAMA) of the Brazilian Ministry of Environment under ABIO N° 755/2016. The whale was found stranded alive on October 19th, 2020, in a beach (−28.1663385: −48.6577602), located on the city of Imbituba, in Santa Catarina state, Southern Brazil. The animal was immediately monitored in the water by the Santos Basin Beach Monitoring Project team. External examination revealed multiple signs of interspecific interaction in the ventral and lateral regions of the body and a deep circular lesion consistent with bite marks from cookiecutter sharks (*Isistius* spp.). The whale was carefully monitored during transport, receiving benzodiazepines (Diazepam, 0.1 mg/kg, IM) and bronchodilators (Aminophylline, 4 mg/kg, IM, SID) to dilate the lungs’ airways. Upon arrival at Associação R3 Animal, in Florianópolis, the whale was transferred to a 60,000 L tank under continuous supervision. To replace the animal’s hydration, 1.5 L of water were initially administered through a 17 mm equine nasogastric tube and later the volume was increased to 3 L every 3 h and antibiotic therapy was started (Enrofloxacin, 5 mg/kg, IM, BID). On October 21, the animal begun to show signs of agitation, swimming erratically, jumping, and bending its body, even after being sedated. The veterinary staff observed prolonged periods of apnea and bradycardia. Cardiac massage and administration of emergency drugs were attempted, but the animal succumbed to death.

At necropsy, besides other gross alterations, the prescapular lymph nodes were clearly swollen. A yellowish purulent material was collected from the lymph nodes and placed in Stuart transport medium, and immediately sent to a microbiology laboratory for bacterial culture and antimicrobial susceptibility testing. The *E. coli* strain (BA01) was recovered, being identified by matrix-assisted laser desorption ionization–time of flight mass spectrometry (MALDI-TOF MS). Antimicrobial susceptibility was performed and interpreted according to the Clinical and Laboratory Standards Institute recommendations (CLSI, 2020). In this respect, human and veterinary antibiotics were tested, including amoxicillin-clavulanic acid, ceftazidime, cefotaxime, ceftriaxone, ceftiofur, cefepime, cefoxitin, aztreonam, ertapenem, meropenem, imipenem, ciprofloxacin, levofloxacin, trimethoprim/sulfamethoxazole, fosfomycin, gentamicin, tetracycline, and amikacin. A drug-susceptible *E. coli* (ATCC 25922) was included as a control strain. Moreover, to determinate whether BA01 strain could survive in the marine environment, tolerance test of NaCl minimal inhibitory concentration (MIC) was performed using 0.1–15% NaCl solutions (Fernandes et al., 2020a).

### Whole Genome Sequence Analysis

**FIGURE 1:**
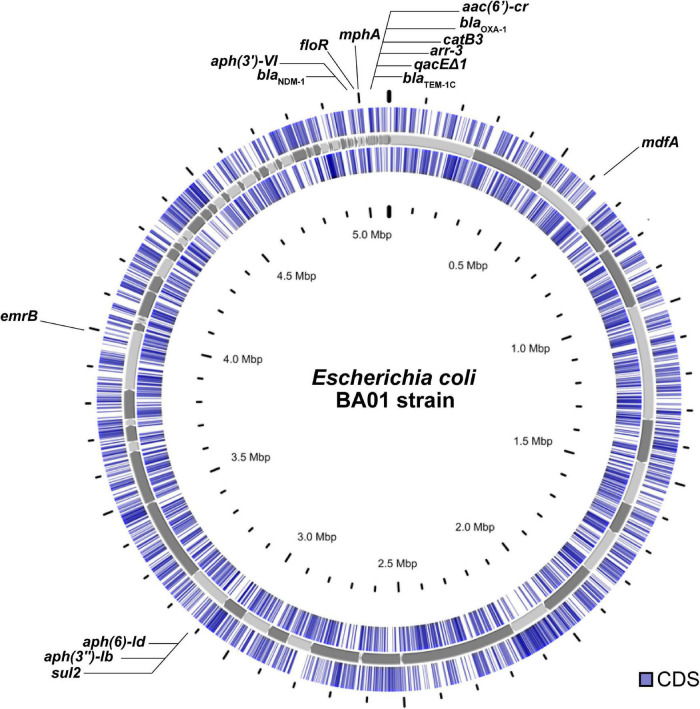
Circular genome view of the *Escherichia coli* BA01 strain.

The whole genomic DNA of *E. coli* BA01 was extracted (PureLink™; Invitrogen) and used to prepare a library that was sequenced using the NextSeq550 platform (2 × 75-bp paired-end) (Illumina). Raw sequencing data were quality filtered to remove low-quality bases (Phred20 quality score) using Trimmomatic v0.32.^1^ The sequence reads were assembled *De novo* using default parameters of Unicycler v0.4.8.^2^ Draft genome sequence was automatically annotated using the NCBI Prokaryotic Genome Annotation Pipeline v.3.2.^3^ BA01 circular genome map (Figure 1) was performed using the Proksee platform^4^ and BLASTN.

Multilocus sequence type (MLST), plasmid replicons, resistome, virulome, type fimbrial, and serotype were performed *in silico* using MLST v2.0, PlasmidFinder v2.0, ResFinder 4.0, VirulenceFinder 2.0, FimTyper v1.0, and SerotypeFinder v2.0, respectively; available from the centre for Genomic Epidemiology.^5^ In addition, ABRicate v0.9.8^6^ was used to screen putative virulence factors through VFDB database.^7^

The presence of heavy metal (HM) genes was predicted by comparison with the BacMet—Antibacterial Biocide and Metal Resistance Genes Database,^8^ whereas for detection of mercury, arsenic, and disinfectant resistance genes DRG (quaternary ammonium compounds), we performed alignment of sequenced reads against our in-house database. Moreover, the presence of halotolerance-associated genes (*katE* and *nhaA*) was *in silico* investigated using BLASTN. A ≥ 90% identity threshold was used as a filter for all accessed databases.

### Phylogenetic Analysis

In order to compare BA01 with other *E. coli* strains, we performed a search for *E. coli* ST162 on *Escherichia*/*Shigella* database in Enterobase.^9^ For phylogenetic analysis purpose, FastANI v1.32^10^ was used to select the 30 genomes with highest average nucleotide identity (ANI) to BA01 among 542 genome assemblies of strains with data for country, year, and source of isolation downloaded from Enterobase. CSI phylogeny v1.4^11^ was used with default settings to generate a maximum-likelihood phylogenetic tree with BA01 and the 30 selected genomes. *E. coli* ST162 strain W2-5 chromosome sequence (RefSeq accession number NZ_CP032989.1) was used as reference. ABRicate v1.0.1 (see text footnote 6) was used with ResFinder and PlasmidFinder databases to screen the genomes for antimicrobial resistance and plasmid replicons. Identity and coverage were set to 98 and 100%, respectively. Mutations in quinolone resistance-determining regions were assessed using CGE PointFinder pipeline.^12^ iTOL v6^13^ was used to root the tree at midpoint and to annotate the tree with Enterobase and ABRicate data.

### *In vivo* Virulence Assays in the *Galleria mellonella* Infection Model

To evaluate the virulence potential of strains, an *in vivo* experiment was carried out with the *Galleria mellonella* infection model (Tsai et al., 2016; Moura et al., 2018). *G. mellonella* larvae, of nearly 250–350 mg, were inoculated with 10^5^ CFU of each strain and survival analysis was evaluated each hour, for 96 h. For each strain, groups of *G. mellonella* containing five larvae were evaluated. *E. coli* strain ATCC 25922 was used as non-virulent control, whereas hypervirulent meningitis/sepsis-associated K1 *E. coli* MNEC RS218 strain was used as hypervirulent control samples (Fuentes-Castillo et al., 2021a). Data were analyzed by the log rank test, with P of 0.05 indicating statistical significance (Graph Pad Software, San Diego, CA, United States).

### Plasmid Conjugation

To evaluate the transferability of the *bla*_NDM–1_ gene, conjugation experiments were carried out. Plasmid conjugation was assessed by mating-out assay using *E. coli* BA01 and sodium azide-resistant *E. coli* C600 (lactose-negative) as donor and recipient strains, respectively. Transconjugants were obtained from MacConkey agar plates supplemented with ertapenem (4 μg/mL) and sodium azide (100 μg/mL).

## Results and Discussion

The *E. coli* strain BA01 strain displayed a MDR profile to amoxicillin/clavulanic acid, cefotaxime, ceftriaxone, cefepime, cefoxitin, ceftiofur, ertapenem (MIC = 16 mg/L), imipenem (MIC = 16 mg/L), meropenem (MIC = 16 mg/L), amikacin, ciprofloxacin, enrofloxacin, levofloxacin, chloramphenicol, and tetracycline (Magiorakos et al., 2012), remaining susceptible to aztreonam, gentamicin, sulfamethoxazole/trimethoprim, and fosfomycin. Additionally, BA01 strain displayed NaCl tolerance (>10%), confirming its ability to survive in the marine environment.

Genomic analysis revealed a broad resistome, with genes conferring resistance to β-lactams (*bla*_NDM–1_, *bla*_TEM–1C_, and *bla*_OXA–1_), aminoglycosides [*aph(6)-Id*, and *aph(3″)-Ib*, *aph(3′)-VI*], macrolide, (*ermB*, *mdfA*, and *mphA*), rifamycin (*arr-3*), quinolones [*aac(6′)-Ib-cr*, and *qnrB6*], phenicols (*catB3* and *floR*), sulfonamide (*sul1* and *sul2*), and tetracycline (*tetA*) (Table 1). Additionally, chromosomal point mutations in ParC (S80I) and GyrA (S83L and D87N) were detected, which may justify the fluoroquinolone-resistant profile. Furthermore, plasmid replicons IncFIB and IncA/C2 were also detected (Table 1).

**TABLE 1:** Genomic and epidemiological data of *E. coli* strain BA01 isolated from a pygmy sperm whale (*Kogia breviceps*) in Brazil.

Strain	BA01
Genome size (Mbp)	5.7
No. of CDS^a^	4,744
tRNA (*n*)	56
rRNA (*n*)	71
Non-coding RNA (*n*)	11
Pseudogenes	136
CRISPR	2
MLST (ST)^b^	162
Resistome	
β-lactams	*bla*_NDM–1_, *bla*_*TEM*–1C_, *bla*_*OXA*–1_
Aminoglycosides	*aph(6)-Id, aph(3″)-Ib, aph(3′)-VI*
Fluoroquinolones	*aac(6′)-Ib-cr, qnrB6, gyrA* (S83F, D87A), *parC* (S80I)
Tetracyclines	*tet(A)*
Rifamycins	*arr-3*
Phenicols	*catB3*, *floR*
Sulphonamides	*sul1*, *sul2*
Macrolides	*ermB, mdf, mphA*
Heavy metal and Biocides	*acrEF, arsBCR, emrDK, mdtEFKN, mvrC, phnCDGHIJKLMNOP, tehAB, tolC, yjiO*
Halotolerance genes	*katE, nhaA*
Virulome	*astA, entA, entC, entE, entB, entD, entF, entS, csgB, csgD, csgF, csgG, espX4, espX5, fdeC, fepA, fepB, fepC, fepD, fepG, fes, espL1, espR1, fimA, fimB, fimC, fimD, fimE, fimF, fimG, fimH, fimI, gspC, gspD, gspE, gspF, gspG, gspH, gspI, gspJ, gspK, gspL, gspM, espX1, iroB, iroC, iroD, iroE, iroN, iucA, iucB, iucC, iucD, iutA, ompA, ykgK/ecpR, yagZ/ecpA, yagY/ecpB, yagX/ecpC, yagW/ecpD, yagV/ecpE*
Plasmidome	IncC-ST3, IncFIB [F18:A-:B1]
GenBank accession number	JAENJJ000000000
OneBR ID	ONE128

*^a^CDSs, coding sequences.*

*^b^MLST, Multilocus sequence type. ST, sequence type.*

Halotolerance associated genes *katE* and *nhaA*, which encodes for catalase and Na + /H + antiporter cytoplasmic membrane, respectively, were *in silico* predicted (Rimon et al., 2007; Prodhan et al., 2008). Furthermore, genes conferring resistance to heavy metals [i.e., arsenic resistance (*arsBCR*), tellurite (*tehAB*)] and biocides [i.e., quaternary ammonium compounds (*acrEF*, *emrK*, *mdtEFKN, mvrC, tolC, yjiO*) and glyphosate (*phnCDEFGHIJKLMNOP*)] were also detected (Table 1).

**FIGURE 2:**
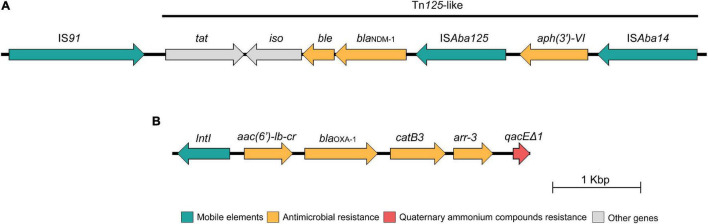
Schematic presentation of the genetic environment context of the *bla*_NDM–1_ gene **(A)** and the class I integron **(B)** identified in the *Escherichia coli* BA01 strain. Arrows indicate protein-coding sequences and are colored by function.

The *bla*_NDM–1_ gene was located on the IncC plasmid and was successfully transferred to the *E. coli* C600 strain, being confirmed by PCR-based replicon typing of transconjugant (Carattoli et al., 2005). The transconjugant *E. coli* displayed resistance to amoxicillin/clavulanic acid, cefotaxime, ceftriaxone, cefepime, cefoxitin, ceftiofur, ertapenem, imipenem, meropenem, amikacin, ciprofloxacin, enrofloxacin, levofloxacin, chloramphenicol, and tetracycline, remaining susceptible to aztreonam, gentamicin, sulfamethoxazole/trimethoprim, and fosfomycin. However, due to limitations of short-read sequencing technology, it was not possible to obtain complete nucleotide sequences of this plasmid. Further analysis revealed that the aminoglycoside 3′-phosphotransferase [*aph(3*′*)-VI*] and the carbapenemase-encoding *bla*_NDM–1_ genes were located downstream of IS*Aba125* and IS*Aba14* mobile genetic elements, respectively, along with the bleomycin resistance protein (ble_MBL_), N-(5′-phosphoribosyl)anthranilate isomerase (*iso*) and twin-arginine translocation pathway signal protein (*tat*) being harbored by a Tn*125*-like transposon (Figure 2A) identified in a 8,630-bp contig highly similar (100% nucleotide identity; 100% query coverage) to that found on *Klebsiella pneumoniae* plasmids (Genbank accession number: LR697132.1; LR697099.1; CP021961.1) and close related (100% nucleotide identity; 70% query coverage) to pAB17 plasmid (Genbank accession number: MT002974.1) identified in a nosocomial lineage of *Acinetobacter baumannii* in Brazil (Rossi et al., 2021). In addition, we also identified a class 1 integron carrying an integron-integrase gene (*intI1*) along with other genes encoding antimicrobial resistance, including aminoglycoside-6′-N-acetyltransferase-Ib [*aac(6*′*)Ib-cr*], class D beta-lactamase OXA-1 (*bla*_OXA–1_), chloramphenicol O-acetyltransferase (*catB3*), rifampin ADP-ribosyl transferase (*arr-3*), and quaternary ammonium compound (*qacE*Δ*1*) (Figure 2B). In this respect, there is a growing concern about the spread of biocides contaminating aquatic environments, especially QACs, since these compounds are widely used in domiciliary and hospital setting, including disinfectants formulations (Zubris et al., 2017). As a consequence, ecosystems impacted by heavy metal and biocides could favor the selection and persistence of MDR bacteria harboring broad resistomes (Baker-Austin et al., 2006; Kim et al., 2018).

*E. coli* ST162 is a pandemic lineage that has been isolated from multiple sources including clinical, environmental, and domestic and wild animal samples (Fuentes-Castillo et al., 2020). When compared with BA01, the 30 selected *E. coli* ST162 genomes for the phylogenetic tree had ANI ranging between 99.7994 and 99.8948%. Among the 31 genomes analyzed, SNP counts variated between 0 and 1,343 (Supplementary Table 1).

**FIGURE 3:**
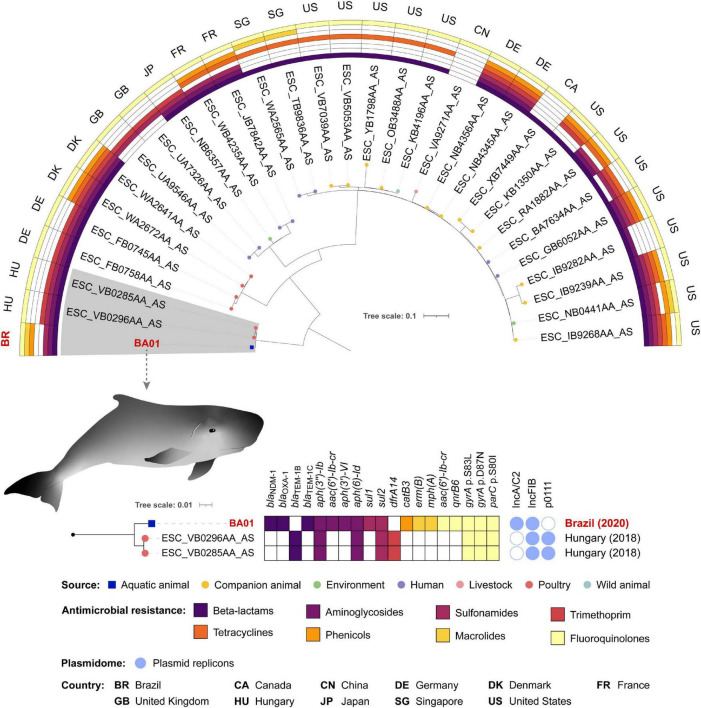
Phylogenetic tree of 31 *Escherichia coli* ST162 strains, plotted in a 180° arc, as well as their predicted phenotype for antimicrobial resistance, source and country of isolation. The highlighted clade with 3 strains is shown in a subtree with resistome, plasmidome, country and year of collection of each isolate.

Phylogenetic analysis revealed that BA01 is closely related to two strains isolated in 2018 from poultry in Hungary, differing from both strains by 59 SNPs (Figure 3 and Supplementary Table 1). While these two strains from Hungary share the same resistome, BA01 has several resistance genes that are absent in these strains [*bla*_NDM–1_, *bla*_*OXA*–1_, *bla*_*TEM*–1C_, *aac(6*′*)-lb-cr*, *aph(3*′*)-VI*, *sul1*, *catB3*, *erm(B)*, *mph(A)*, and *qnrB6*], as well as an IncC-type plasmid.

**FIGURE 4:**
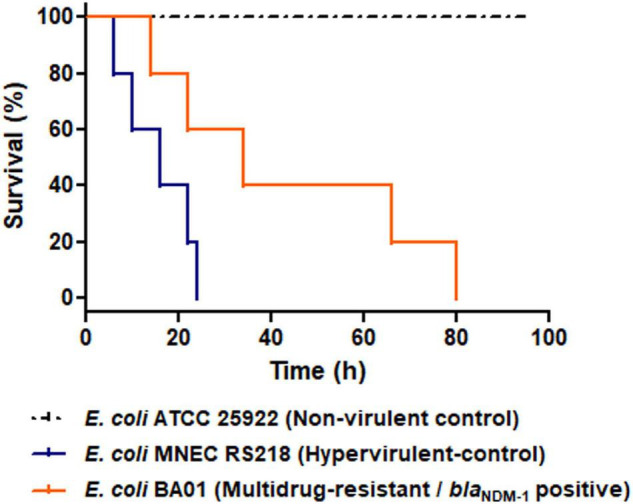
Virulent behavior of NDM-1-positive *Escherichia coli* isolated from a pygmy sperm whale (*Kogia breviceps*), in Brazil. Kaplan-Meier survival curves of *G. mellonella* infected with 10^5^ CFU/larvae of the NDM-1-positive *E. coli* BA01 strain (orange line), the non-virulent *E. coli* ATCC 25922 strain (dashed black line) and the hypervirulent meningitis/sepsis associated *E. coli* strain MNEC RS218 strain (blue line). The *E. coli* BA01 strain and the hypervirulent meningitis/sepsis-associated K1 *E. coli* MNEC RS218 strain killed 70 and 100% of the *G. mellonella* larvae within 80 h post-infection, respectively, leading to higher mortality rates than the non-virulent *E. coli* ATCC 25922 strain (*P* < 0.05). For each strain, groups of *G. mellonella* containing five larvae were evaluated in three separate experiments.

Virulome of BA01 strain, included genes/operons that encodes to enteroaggregative EAST-1 heat-stable toxin (*astA*), iron acquisition systems (*entACEDFS*, *fepABCDG*, *fes*, *iroBCDEN*, *iucABCD*, and *iutA*), adherence factors (*fdeC*, *ecpRABCDE*, *csgBDFG*, and *fimABCDEFGHI*), secretion systems components (*espL1*, *espR1*, *espX1*, *espX4*, *espX5*, and *gspCDEFGHIJKLM*) and outer membrane protein A (*ompA*). Of note, the *astA* virulence factor has been commonly found in *E. coli* strains associated with extra-intestinal disease in animals and outbreaks of diarrhea in humans and animals worldwide (Zajacova et al., 2012; Silva et al., 2014; Maluta et al., 2016; Ochi et al., 2017; Dubreuil, 2019). Indeed, the presence of *astA* gene along with other virulent associated genes detected in the BA01 genome (i.e., genes encoding for adherence factors and iron acquisition systems) could favor the virulent behavior of this strain (Fuentes-Castillo et al., 2020, Fuentes-Castillo et al., 2021a), which was supported by *in vivo* experiments using *G. mellonella* larvae. In this respect, the *E. coli* BA01 strain and the hypervirulent meningitis/sepsis-associated K1 *E. coli* MNEC RS218 strain killed 70 and 100% of the *G. mellonella* larvae within 80 h post-infection, respectively, presenting higher mortality rates than the non-virulent *E. coli* ATCC 25922 strain (*P* < 0.05) (Figure 4).

The *bla*_NDM–1_ gene was firstly reported in *Klebsiella pneumoniae* and *E. coli* recovered from a patient in Sweden that was transferred from a New Delhi hospital in 2008 (Yong et al., 2009). Since then, NDM-type carbapenemases have triggered global attention due to their rapid epidemiologic expansion among Enterobacterales and *Acinetobacter* spp., and more rarely, in *Pseudomonas aeruginosa* (Dortet et al., 2014). Of note, recent reports have documented the spreading of NDM producers beyond the boundary of human healthcare settings where they were originally related (Ranjan and Thatikonda, 2021). The environmental spread of NDM-producing bacteria has been associated to several human activities that result in chemical and microbial pollution mostly in aquatic environments (Ranjan and Thatikonda, 2021).

Particularly for marine environments, it has been demonstrated that anthropogenic pollution by improper discharge of effluents from hospitals, domestic sewage, and industrial, urban and/or agricultural wastewaters can runoff to ocean carrying MDR bacteria, antibiotic-resistant genes (ARGs), and heavy metals (Hatosy and Martiny, 2015; Li et al., 2020; Zhang et al., 2022). While it has been suggested that beaches and coastal waters from urbanized and densely populated coastlines are more prone to be contaminated by WHO critical priority bacteria, ocean currents and migratory animals can also favor the spread of these pathogens through long distances, sometimes reaching remote geographical areas with limited human footprints such Polar regions (Hernández and González-Acuña, 2016; Akhil Prakash et al., 2021) and inhospitable oceanic islands (Ewbank et al., 2022).

In this investigation, we report the occurrence of a carbapenem-resistant NDM-1- producing *E. coli* isolated from a pygmy sperm whale. In this regard, the pygmy sperm whale is a small cetacean from the *Kogiidae* family that is found in mesopelagic regions near the continental shelves (between 600 and 1,200 m depth) of the tropical and temperate Atlantic, Indian, and Pacific Oceans (Moura et al., 2016; Brentano and Petry, 2020; Kiszka and Braulik, 2020). Although cetacean research in oceanic waters has significantly progressed over the last decades, there is scarce information on the population, distribution, and behavior of pygmy sperm whales (Kiszka and Braulik, 2020). This could be explained by their short surfacing interval, cryptic surface behavior, and long deep dives, which make challenging to see these whales in the ocean (Kiszka and Braulik, 2020). Indeed, most data come from stranded animals, being generally affected by anthropogenic material, including accidental ingestion of plastic debris (Brentano and Petry, 2020). Alarmingly, increasing reports of WHO critical priority Gram-negative pathogens (MCR-type, carbapenemase- and/or ESBL-producing bacteria) on the Brazilian coast have been occurred in the last decade, which may indicate, in part, the adaptation of such pathogens in the sea. In this regard, the occurrence of such bacteria was documented in coastal waters from in densely coastal areas (Montezzi et al., 2015; Campana et al., 2017; Fernandes et al., 2017, 2020a; Paschoal et al., 2017; Sellera et al., 2017a; Corrêa et al., 2021; Furlan et al., 2021; Cordeiro-Moura et al., 2022), in marine fishes (Sellera et al., 2018a) and benthic invertebrates (Sellera et al., 2018b; Monte et al., 2019; Fernandes et al., 2020b), and also infecting penguins (Sellera et al., 2017b; Wink et al., 2021), a sea turtle (Goldberg et al., 2019), and a dolphin (Fuentes-Castillo et al., 2021b). More specifically, the presence of NDM-1-producing bacteria have been so far identified in *K. pneumoniae* and *Acinetobacter chengduensis* from coastal waters of Rio de Janeiro (Campana et al., 2017; Paschoal et al., 2017; Corrêa et al., 2021), whereas a single case of *E. coli* carrying *bla*_NDM–1_ infecting a penguin was also documented in the South coast of Brazil (Wink et al., 2021).

## Conclusion

In summary, we report for the first time the occurrence of the NDM-1-producing *E. coli* ST162 clone in a marine cetacean. Our findings are worrisome because may indicate that NDM-producing *E. coli* can spill over from the human clinical context to the aquatic environment reaching marine animals with serious clinical implications in wildlife with a further threat to marine ecosystem maintenance. Indeed, recent studies have already demonstrated that WHO critical priority *E. coli* may display halotolerant behavior (Fernandes et al., 2020a), which could favor their spread and persistence in the marine environment. Considering that marine cetaceans are usually found in nearshore waters, exposure to critical priority carbapenemase-producing bacteria could emerge as a new challenge for the conservation of these threatened species. Last but not least, strengthening the epidemiological surveillance of antimicrobial resistance in the ocean is crucial to understanding the ecological implications of these bacteria on marine populations.

## Data Availability Statement

The datasets presented in this study can be found in online repositories. The names of the repository/repositories and accession number(s) can be found in the article/Supplementary Material.

## Ethics Statement

The animal study was reviewed and approved by the Brazilian Institute of the Environment and Renewable Natural Resources (IBAMA) of the Brazilian Ministry of Environment under ABIO N°755/2016; all animal handling procedures and protocols followed the required ethics and welfare practices.

## Author Contributions

FS, BC, DF-C, BF, ES, and FE performed data analysis. FS, BC, DF-C, BF, ES, FE, and HF conducted the experiments. FS, BC, DF-C, BF, ES, FE, DG, and NL wrote the manuscript. NL designed, coordinated the project, and supervised. MA, LS, SS, CK, and NL reviewed and edited the manuscript. All authors have read and agreed to the published version of the manuscript.

## Conflict of Interest

The authors declare that the research was conducted in the absence of any commercial or financial relationships that could be construed as a potential conflict of interest.

## Publisher’s Note

All claims expressed in this article are solely those of the authors and do not necessarily represent those of their affiliated organizations, or those of the publisher, the editors and the reviewers. Any product that may be evaluated in this article, or claim that may be made by its manufacturer, is not guaranteed or endorsed by the publisher.

## Footnotes

1
https://github.com/timflutre/trimmomatic

2
https://github.com/rrwick/Unicycler

3
www.github.com/tseemann/prokka

4
https://beta.proksee.ca/

5
http://genomicepidemiology.org/

6
https://github.com/tseemann/abricate

7
https://github.com/haruosuz/vfdb

8
http://bacmet.biomedicine.gu.se

9
https://enterobase.warwick.ac.uk

10
https://github.com/ParBLiSS/FastANI

11
https://cge.cbs.dtu.dk/services/CSIPhylogeny/

12
https://bitbucket.org/genomicepidemiology/pointfinder

13
https://itol.embl.de
