# Supplementary material for: Genomic Analysis of a Highly Virulent NDM-1-Producing Escherichia coli ST162 Infecting a Pygmy Sperm Whale (Kogia breviceps) in South America
Source: Front Microbiol. 2022 Jun 10;13:915375. doi: 10.3389/fmicb.2022.915375 (PMC9231830; doi:10.3389/fmicb.2022.915375)
Supplement: Supplementary file 1 [file Data_Sheet_1.docx]

Supplementary Material

**
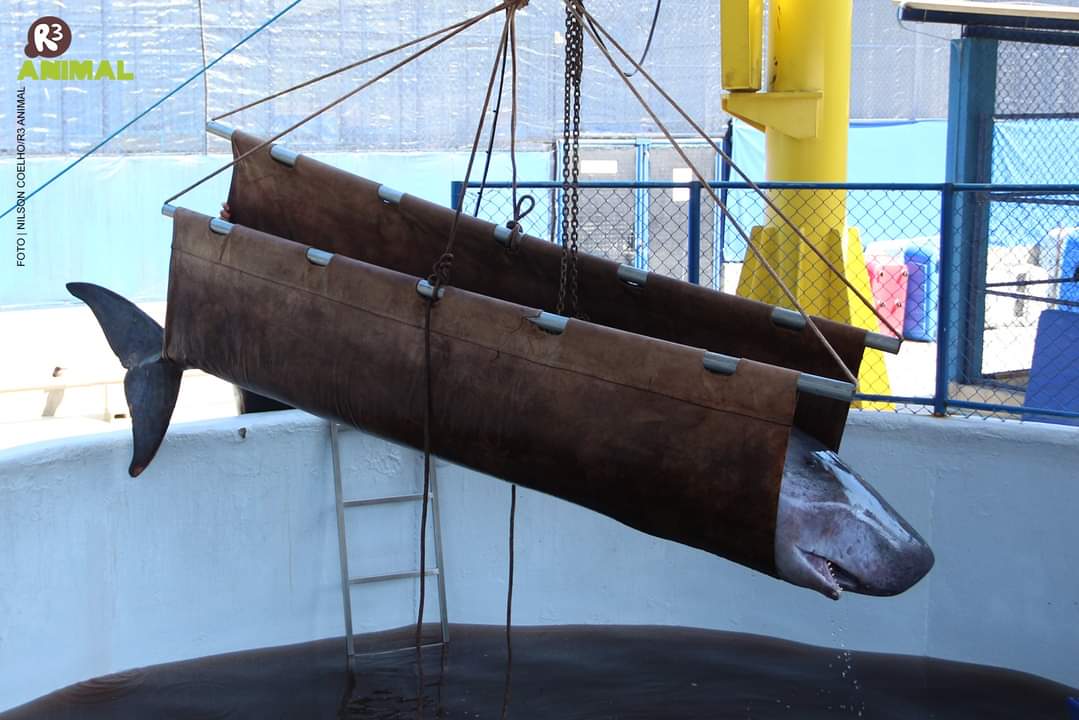
**

**Supplementary Figure 1.** Pygmy sperm whale (*Kogia breviceps*) found stranded alive in Santa Catarina state, South Brazil. The whale was carefully monitored during transport and transferred to a 60.000 liters tank under continuous supervision. A sample of prescapular lymph node was positive for NDM-1-producing *Escherichia coli* (BA01 strain).
